# Male-specific hepatitis B virus large surface protein variant W4P potentiates tumorigenicity and induces gender disparity

**DOI:** 10.1186/s12943-015-0303-7

**Published:** 2015-02-03

**Authors:** Seoung-Ae Lee, Hong Kim, You-Sub Won, Seung-Hyeok Seok, YiRang Na, Han-Bo Shin, Kyung-Soo Inn, Bum-Joon Kim

**Affiliations:** Department of Microbiology and Immunology, Liver Research Institute, Cancer Research Institute and SNUMRC, College of Medicine, Seoul National University, Seoul, Korea; Department of Pharmaceutical Science, College of Pharmacy, Kyung Hee University, Seoul, Korea

**Keywords:** PreS1 mutation, Hepatocellular carcinoma, Estrogen, Interleukin-6

## Abstract

**Background:**

The underlying mechanisms of carcinogenesis and gender disparity in hepatitis B virus (HBV)-induced hepatocellular carcinoma (HCC) remain unclear. Recently, we reported a novel HCC-related W4P/R mutation in the large surface protein (LHB) of HBV genotype C, which was found exclusively in male HCC patients.

**Methods:**

LHB sequences from a carrier (wild type; WT) and W4P variant LHB sequence from an HCC patient were cloned and used to generate NIH3T3 and Huh7 cell lines. Cell proliferation and *in vitro* tumorigenicity were assessed by cell growth and transformation assays. Male and female nude mice were injected with the cells to determine *in vivo* tumorigenicity. To confirm the effect of estrogen in W4P-mediated tumorigenicity, male mice were injected with estrogen and challenged with W4P-expressing cells. The serum levels of different cytokines from the mouse model and patients were analyzed by ELISA. A critical role of interleukin (IL)-6 signaling in W4P-mediated tumorigenicity was tested by inhibition of Jak2.

**Results:**

Although both WT and W4P variant LHBs enhanced cell proliferation by regulating the cell cycle and facilitated cell colony formation, the W4P variant demonstrated significantly higher activity. NIH3T3 cells expressing variant LHB, but not the WT, induced tumor in a nude mouse model. Tumor masses produced by variant LHB were significantly larger in male than female mice, and significantly reduced by estrogen. IL-6, but not tumor necrosis factor-α, was elevated in male mice harboring W4P-induced tumor, and was reduced by estrogen. IL-6 levels of HCC patients with the W4P variant were significantly higher than those of patients with WT LHB. W4P LHB induced higher production of IL-6 than WT LHB in cell lines, and the level was reduced by estrogen. The ability to reduce cell proliferation and colony formation of W4P LHB was hampered by inhibition of IL-6 signaling.

**Conclusions:**

This study suggests that the W4P mutation during the natural course of chronic hepatitis B infection may contribute to HCC development, particularly in male patients, in an IL-6-dependent manner.

**Electronic supplementary material:**

The online version of this article (doi:10.1186/s12943-015-0303-7) contains supplementary material, which is available to authorized users.

## Background

Hepatitis B virus (HBV) infection is a global health problem and >350 million people are chronic carriers of the virus [1,2]. The infection is associated with a wide spectrum of clinical manifestations, ranging from acute or fulminant hepatitis to various forms of chronic infection, including asymptomatic carrier status, chronic hepatitis, cirrhosis, and hepatocellular carcinoma (HCC) [3,4].

Owing to the successful development of HBV vaccine, chronic HBV infection in children has been dramatically reduced worldwide [5,6]. However, adult HBV carriers still have a significant risk for HCC. In particular, HBV genotype C, which is found throughout Asia, has a higher risk for HCC than other genotypes [7,8]. Although the relationship between HBV infection and HCC development is clear, the mechanism by which HBV facilitates cancer development is not clear. Among the viral factors, HBV X protein (HBx) is closely related to HCC development [9-12].

One noteworthy universal epidemiological trait of HCC is the male predominance, with a male:female ratio range of 1.5–11:1 [1,13]. The male predominance is more pronounced in HBV-related than hepatitis-C-virus-related HCC [14,15]. Both higher androgen levels and more active androgen receptor gene alleles are correlated with an increased risk of HCC among male carriers of hepatitis B surface antigen [16]. Recently, an intriguing interaction between HBx and the androgen axis in promoting male HCC was elucidated [17,18].

In addition to the androgen axis, the possible tumor-protective effect of the estrogen axis for HCC has been suggested. For example, longer exposure to estrogen, by taking oral contraceptives or postmenopausal hormone therapy, in female HBV carriers is associated with a lower risk of HCC [19]. Moreover, in a diethylnitrosamine-induced HCC mouse model, estrogen-receptor-α-mediated inhibition of interleukin (IL)-6 secretion from Kupffer cells was critical in alleviating the carcinogenic process [20,21].

The role of HBV large surface proteins (LHBs) in the biology of the virus has yet to be clarified, but it is suggested that LHBs play a role in virus assembly [22] and attachment to hepatocytes [23]. Some studies have proposed that LHBs with mutations in the preS region contribute to hepatocarcinogenesis through induction of an endoplasmic reticulum stress pathway or by altering the transactivating capacity [24-26], which suggests there may be a disparity in the HCC-inducing capacity between wild-type (WT) LHBs and the variant with mutations associated with disease severity.

Recently, through a molecular epidemiological study, we discovered novel preS1 substitutions (W4P) related to HCC, in which tryptophan changed to proline at the fourth codon from the preS1 start [27]. Notably, it occurred exclusively in male patients. Therefore, we hypothesize that the LHB variant with a W4P mutation may contribute to the male predominance in HCC. To address the hypothesis, we investigated whether an LHB variant with a W4P mutation from an HCC patient would exhibit gender disparity in tumorigenicity.

## Results

### LHB variant with W4P mutation showed enhanced cell proliferating activity and facilitated cell cycle progression

To understand the physiological role of W4P mutation of HBV genotype C, LHB sequences from a carrier (WT) and a variant LHB sequence with W4P mutation (W4P) from an HCC patient were cloned. A total of seven variations between the WT and W4P variant LHB sequences were found. Three variations were located in the pre-S1 region, three in the preS2 region, and two in the S region (Additional file 1: Figure S1).

**Figure 1:**
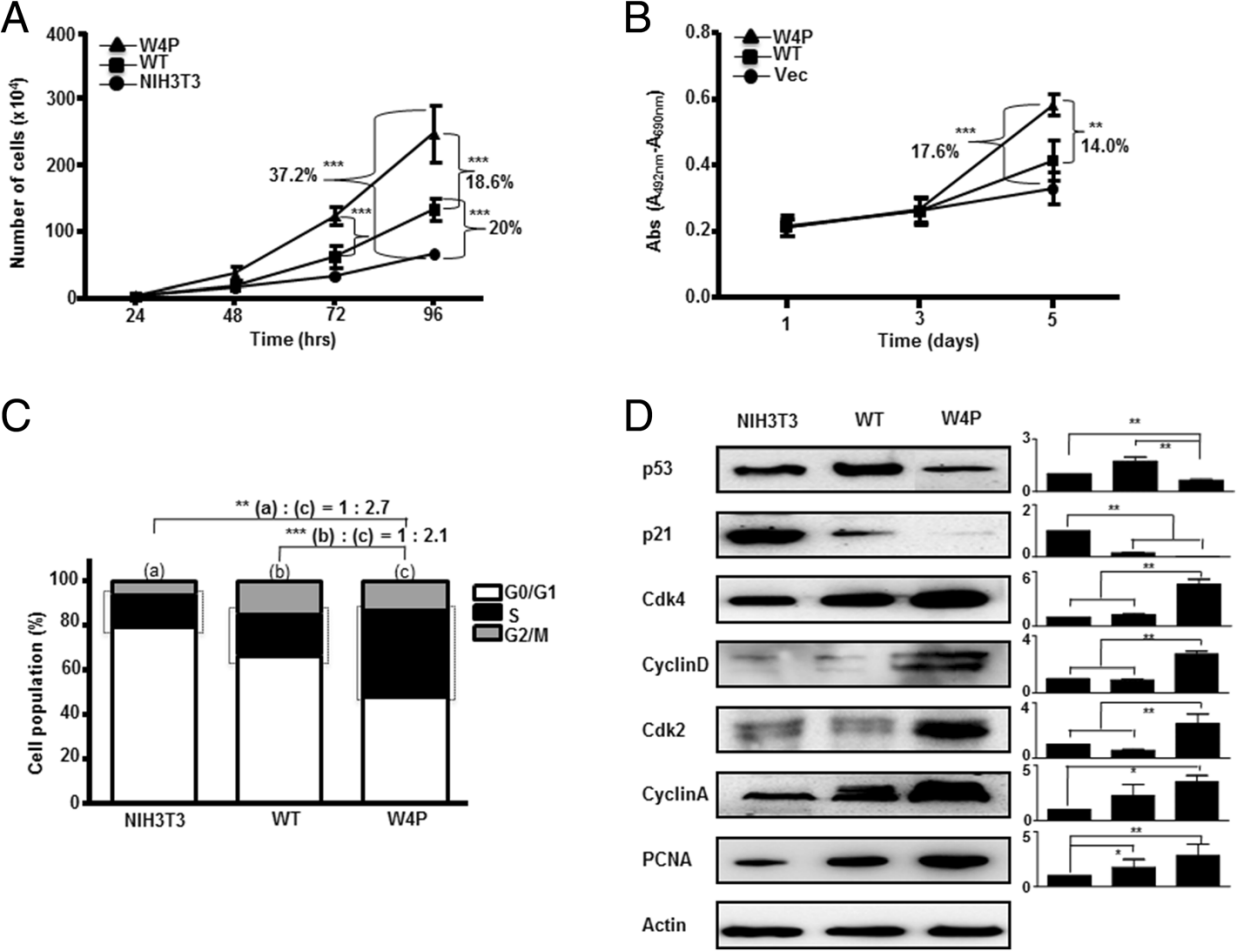
**W4P variant LHB facilitates cell proliferation and cell cycle progression.** **(A and B)** Comparison of growth of WT- and W4P-LHB-expressing NIH3T3 cells **(A)** and Huh7 cells **(B)**. Cell proliferation was analyzed by cell counting. **(C)** Influences on cell cycle regulation by expression of LHBs. The percentage of cells in the G0/G1, S and G2/M phases was assessed by flow cytometry after propidium iodide staining. **(D)** Effect of LHBs on cell-cycle-related proteins. Expression of cell-cycle-related proteins in LHB-expressing cells was analyzed by immunoblotting. R elative amounts of the proteins were analyzed by densitometry using actin as an internal control for normalization (right). The standard deviations are determined from three independent experiments. *P < 0.05, **P < 0.01, ***P < 0.001 versus NIH3T3 cell line, one-way *t* test.

The W4P variation was related to HCC; therefore, we examined whether the variation was implicated in cell growth using mouse NIH3T3 cell lines constitutively expressing WT LHB (WT-LHB-NIH3T3) and W4P variant LHB (W4P-LHB-NIH3T3) (Additional file 2: Figure S2). As shown in Figure 1A, both WT and W4P LHBs significantly facilitated cell proliferation. Notably, W4P LHB showed significantly greater enhancement of cell growth than WT LHB showed. Similar results were obtained from the experiment using Huh7 human HCC cell lines expressing vector, WT and W4P LHBs.

LHBs facilitate cell proliferation; therefore, we investigated whether LHBs regulate cell cycle progression. Expression of WT and W4P LHB induced 33.5% and 52% increases in the percentages of cells in S and G2/M phases, respectively (Figure 1C). The levels of p53 and p21 proteins, which are associated with the G1/S checkpoint, were reduced by expression of LHBs, and W4P exerted a stronger effect than WT (Figure 1D). The levels of cyclin-dependent kinase (CDK)2, CDK4, cyclin A, and cyclin D, which are known to be involved in G1/S transition, were higher in W4P expressing cells than WT and NIH3T3 cell lines (Figure 1D). These results suggest that LHBs, especially W4P LHB, regulate cell cycle progression, and LHBs may affect G1/S transition. An increase in the well-known proliferation marker proliferating cell nuclear antigen (PCNA) by W4P LHB supported the higher cell-proliferating activity of W4P LHB (Figure 1D).

### W4P-LHB-expressing cells had oncogenic potential to form tumor masses in a mouse model and tumor growth showed gender disparity

**Figure 2:**
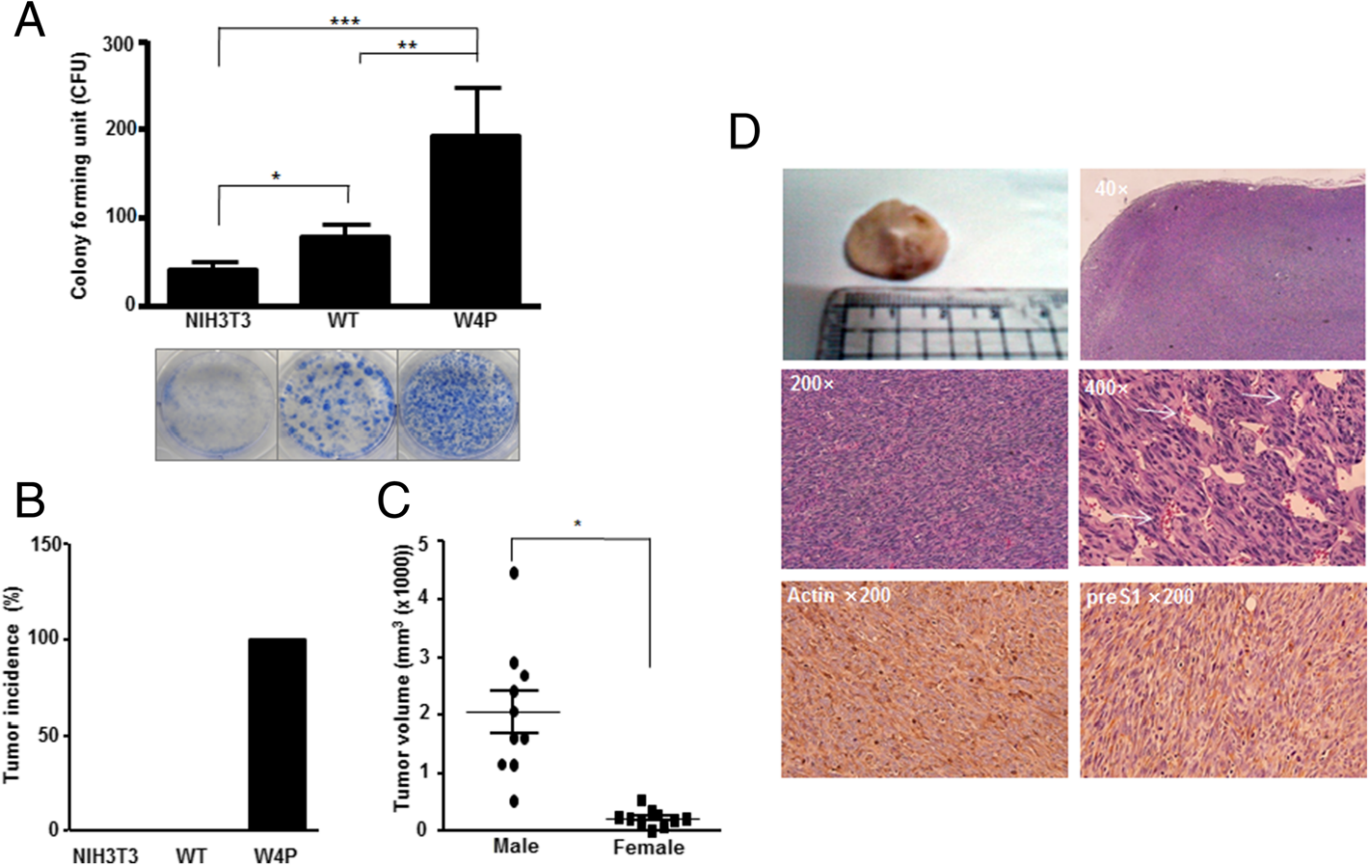
**Transforming activity of W4P variant and gender disparity in tumorigenesis of W4P LHB expressing.** **(A)** Induction of cell transformation by LHBs. NIH3T3 cells and NIH3T3 cell lines expressing WT and W4P LHBs were subjected to a colony formation assay. Data represent means ± SD from three independent experiments. **P < 0.01 versus pIRES2-WT, ***P < 0.001 versus NIH3T3 cell line, one-way *t* test. **(B)** Tumor-forming abilities of LHB-expressing cells. Nude mice were injected with NIH3T3 (male, *n* = 10), WT-LHB-NIH3T3 (WT) (male, *n* = 10) and W4P-LHB-NIH3T3 [male (*n* = 10) and female (*n* = 10)] cells. The graph shows the tumor incidence of each group. **(C)** Comparison between male and female mice in terms of tumor size in nude mice injected with W4P cells. *P < 0.05. **(D)** Hematoxylin and eosin staining of the tumor mass of nude mice injected with W4P cells. Expression of LHB was confirmed by immunohistochemical staining using preS1 Ab (AP1).

We examined whether W4P mutation was involved in cell transformation. W4P-LHB-NIH3T3 cells showed a significantly higher colony forming ability than WT-LHB-NIH3T3 and NIH3T3 cells. Compared with NIH3T3 cells, the colony numbers in WT and W4P increased by about 1.9 and 4.6 times, respectively, suggesting that W4P LHB regulates the cell cycle and has a strong transforming effect on NIH3T3 cells (Figure 2A). Similarly, W4P-LHB-expressing Huh7 cells lines formed increased numbers of colonies compared with vector cells or WT-LHB-expressing cells, suggesting that W4P LHB facilitates growth of HCC cells (Additional file 3: Figure S3).

To test further whether W4P variation was involved in tumorigenicity and gender disparity of HCC, we grafted the cell lines into nude mice. Surprisingly, 19 (10 male and 9 female) of the 20 mice injected with W4P cells developed tumors, whereas no tumors were noted in the groups injected with WT-LHB-NIH3T3 and the control NIH3T3 cells during the 4 weeks observation period, indicating that LHB gained oncogenic potential by W4P mutation (Figure 2B). Although there was no significant difference in tumor incidence between male and female mice, surprisingly, the tumor volume in the W4P-cell-injected male mice was significantly larger than in the female mice (Figure 2C). W4P-LHB-induced tumor showed a typical fasciculated proliferating pattern with an interstitial collagen matrix (Figure 2D). Most areas consisted of highly polymorphic, poorly differentiated spindloid cells with hyperchromatic nuclei. The tumor cells had an oval vesicular nucleus with two instances of prominent nucleoli and basophilic cytoplasm. The mitotic rate was high and apoptotic cells were occasionally found. Most tumor cells expressed the preS1 antigen in their cytoplasm (Figure 2D).

### W4P-LHB-expressing cells induced production of high levels of IL-6, especially in male mice

**Figure 3:**
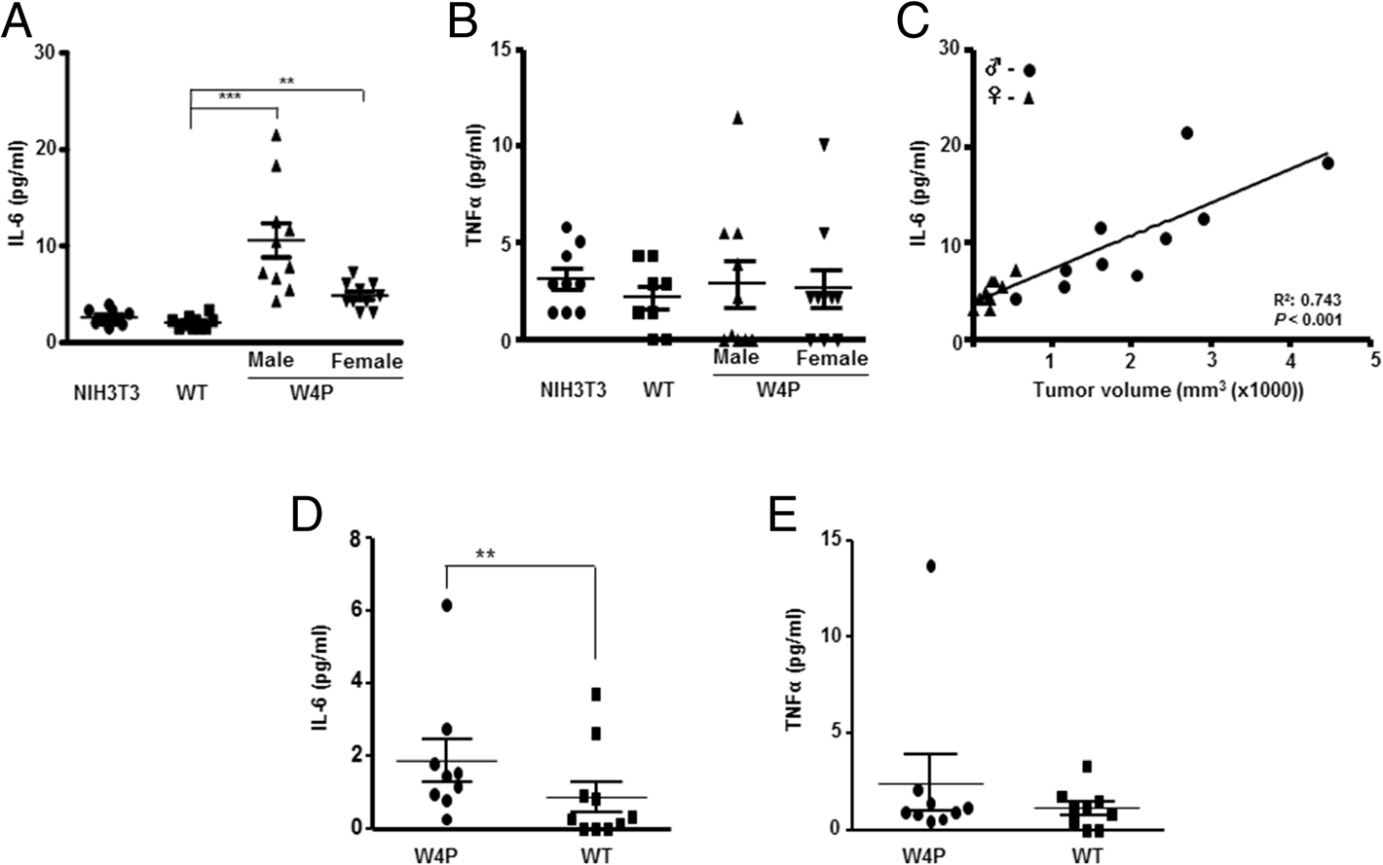
**Induction of IL**-**6 production by W4P LHB in a gender**-**dependent manner.** **(A and B)** Serum levels of IL-6 **(A)** and TNF-α **(B)** measured from mice injected with NIH3T3, WT-LHB-NIH3T3 and W4P-LHB-NIH3T3 cells. **P < 0.01, ***P < 0.001. **(C)** Correlation between serum IL-6 levels and tumor volumes of W4P-LHB-NIH3T3-injected mice (10 male and 10 female mice) (*r* = 0.862, *P* < 0.001). **(D and E)** Serum IL-6 **(D)** and TNF-α **(E)** was measured in 11 HCC patients with WT and 11 with W4P variant sequences. **P < 0.01.

IL-6 is significantly related to gender disparity in hepatocarcinogenesis [20,21]; thus, we attempted to determine whether IL-6 was also related to gender disparity in the tumorigenicity in our W4P-injected mouse model. Serum IL-6 level in the W4P-injected mice was significantly higher than in mice injected with NIH3T3 or WT (Figure 3A), whereas there was no significant difference in the level of tumor necrosis factor (TNF)-α (Figure 3B). Serum IL-6 level of W4P-LHB-NIH3T3-injected mice was significantly higher in male than in female mice, suggesting the involvement of IL-6 in gender disparity of W4P-induced tumor growth (Figure 3A). Supporting this hypothesis, there was a positive correlation between IL-6 serum level and tumor size irrespective of gender (Figure 3C). In line with the mouse data, HCC patients with the W4P variant had significantly higher serum IL-6 levels than those carrying WT LHB (Figure 3D), while there was no significant difference in the level of TNF-α (Figure 3E).

### IL-6 signaling pathway was necessary for cell proliferating and transforming activities of W4P LHB

**Figure 4:**
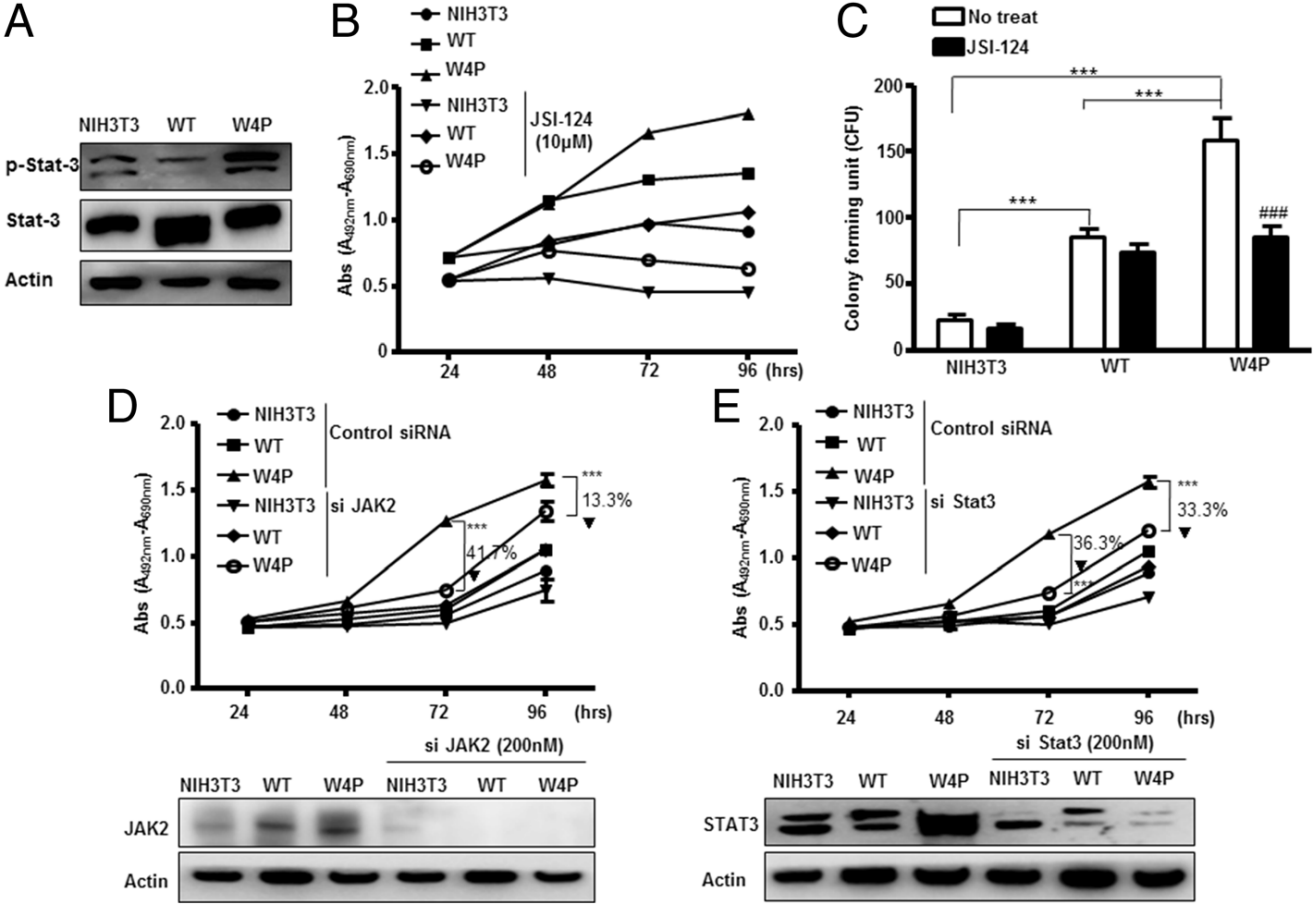
**Effects of inhibition of IL**-**6 signaling on proliferation and colony**-**formation of LHB**-**expressing cells.** **(A)** Phosphorylated stat3, stat3 and actin were analyzed by immunoblotting. **(B)** Designated cells were treated with JSI-124 (10 μM), a Jak2 inhibitor, and their proliferation was assessed. **(C)** Colony-forming ability of LHB-expressing cells was examined by a colony forming assay in the absence or presence of JSI-124. **(D and** **E)** Cells were transfected with siRNAs (200 nM) targeting JAK2 **(D)**, stat3 **(E)** or control scrambled siRNAs as indicated. Cells proliferation was analyzed by MTT assay. Data represent means ± SD. **P < 0.01, ***P < 0.001.

We examined the phosphorylation status of signal transducer and activator of transcription (stat)3, which can be activated by IL-6 receptor (IL-6R)–Janus kinase (JAK)2 signaling. W4P-LHB-expressing NIH3T3 and Huh7 cells displayed higher phosphorylation of stat3 than WT-LHB-expressing cells and parental cells (Figure 4A and Additional file 4: Figure S4). We took advantage of the pharmacological inhibition of the IL-6 signaling pathway by JSI-124, a specific inhibitor of JAK2. Although treatment with JSI-124 reduced the proliferation of both WT- and W4P-LHB-expressing cells, reduction of W4P-LHB-expressing cells was much greater than that of WT-LHB-expressing cells (Figure 4B). The cell transforming activity of W4P was also suppressed by treatment with JSI-124, and there was no significant difference in the transforming activities of WT- and W4P-LHB-expressing cells after treatment with JSI-124 (Figure 4C). Suppression of the JAK2–stat3 signaling axis by siRNAs targeting JAK2 or stat3 resulted in significant suppression of W4P-LHB-expressing cell growth (Figure 4D, 4E).

### W4P-LHB-mediated IL-6 production and tumor growth were downregulated by estrogen

**Figure 5:**
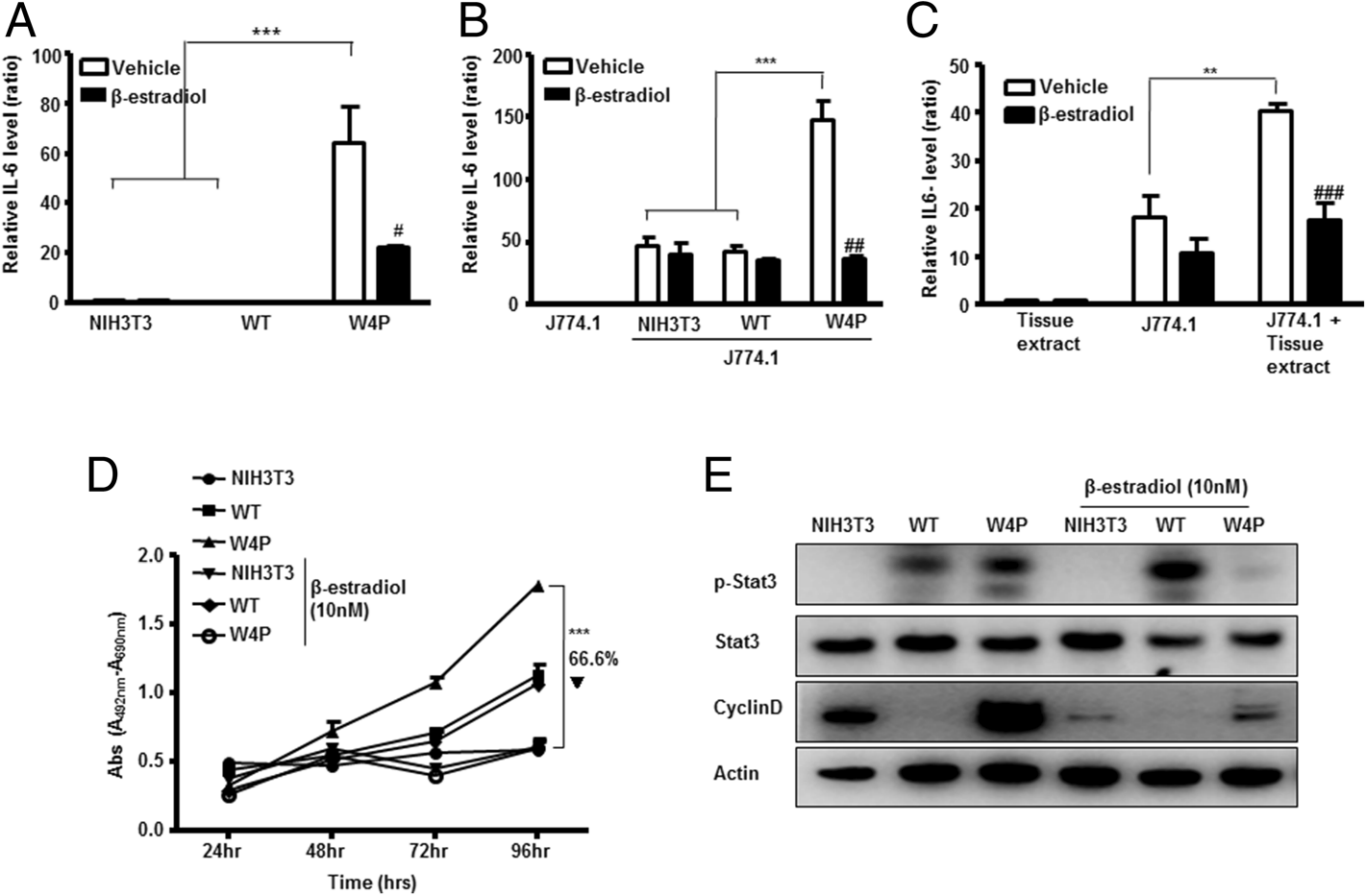
**Effect of estrogen on W4P**-**LHB**-**mediated IL**-**6 production and cell proliferation.** **(A)** Cells were incubated for 48 h with or without 20 nM estradiol. Production of IL-6 was determined by ELISA. **(B)** J774A.1 cells were co-cultivated with indicated cells for 48 h in the presence or absence of 20 nM estradiol. **(C)** J774A.1 cells were treated with 10-mg lysates made from tumors expressing W4P LHB in the presence or absence of 20 nM estradiol. **(D)** Cells were treated with vehicle (0.01% ethanol) or 10 nM β-estradiol and subjected to MTT assays. **(E)** Cells were treated with vehicle (0.01% ethanol) or 10 nM β-estradiol for 48 h and analyzed by immunoblotting to determine the level of stat3 phosphorylation and cyclin D. Data represent means ± SD. *P < 0.05, **P < 0.01, ***P < 0.001.

W4P-LHB-mediated production of IL-6 is involved in tumor growth and IL-6 production was significantly lower in female mice; therefore, we tested the hypothesis that estrogen signaling may suppress IL-6 production and inhibit IL-6-mediated tumor growth. Consistent with the *in vivo* data, W4P-LHB-NIH3T3 cells produced a significant amount of IL-6, while the WT-LHB-NIH3T3 and NIH3T3 cells did not produce a detectable amount of IL-6 (Figure 5A). Treatment with β-estradiol of the W4P-LHB-NIH3T3 cells resulted in a significant decrease of IL-6 production (Figure 5A). Co-cultivation of the murine macrophage cell line J774A with W4P-LHB-NIH3T3 cells resulted in increased production of IL-6, which was reduced by treatment with estradiol (Figure 5B). Secretion of IL-6 by J774 was significantly enhanced by treatment of W4P tumor lysates but not WT tumor lysate, and the level was lowered by estradiol, suggesting that IL-6 is produced by W4P-LHB-expressing cells and neighboring macrophages (Figure 5C). Consistent with the previous data showing the crucial role of IL-6–JAK2–stat3 signaling pathway, treatment with β-estradiol completely abolished the effect of W4P LHB on cell growth (Figure 5D). In addition, β-estradiol treatment suppressed phosphorylation of stat3 in W4P LHB-expressing cells, further supporting the hypothesis that W4P LHB induced higher cell proliferation and tumorigenesis (Figure 5E).

**Figure 6:**
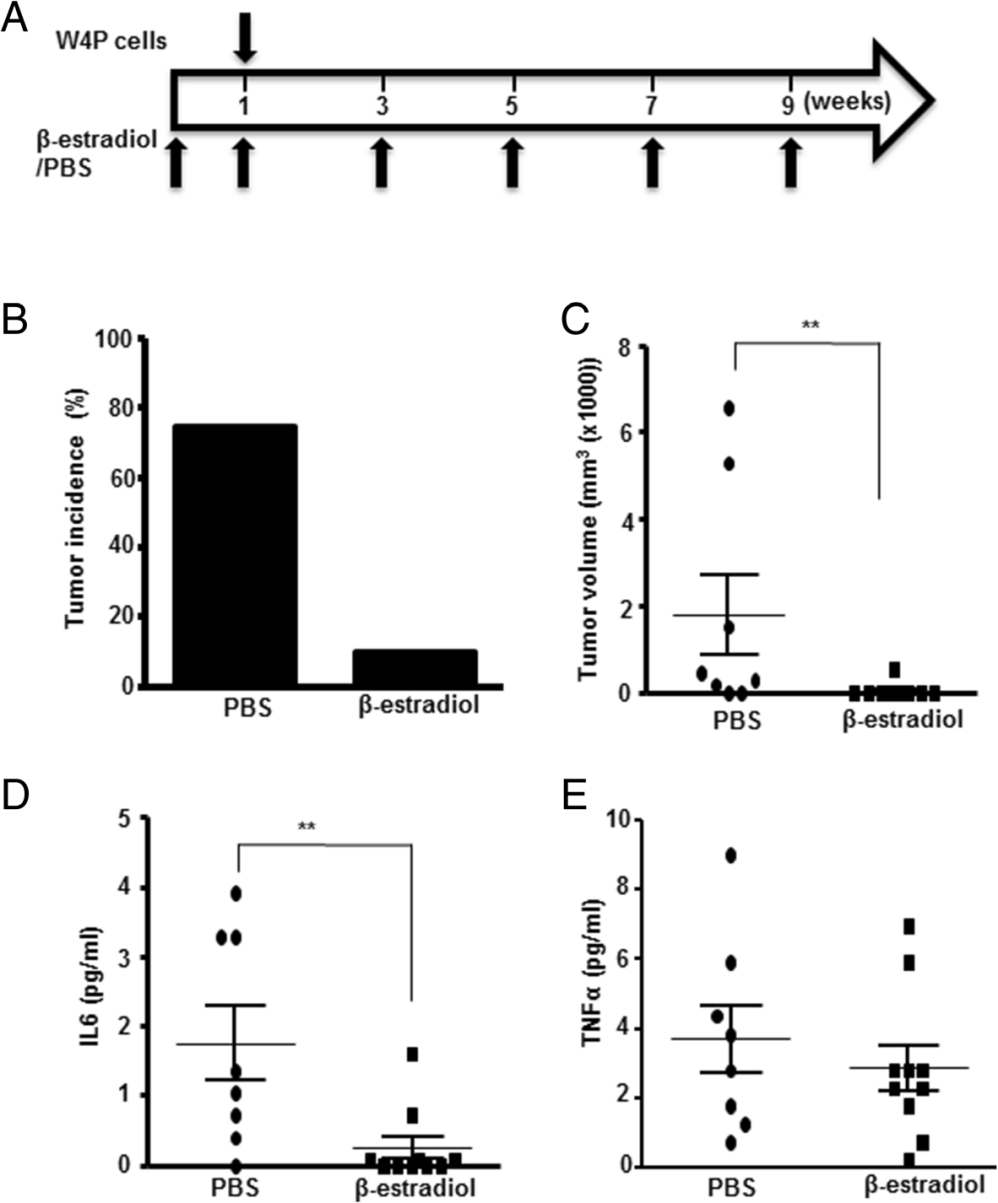
**Suppression of W4P**-**mediated tumorigenicity by estrogen.** **(A)** Scheme of the experiment. Male mice were injected with 0.5 mg/kg β-estradiol or PBS. One week later, mice were injected with W4P-LHB-expressing cells subcutaneously together with 0.5 mg/kg β-estradiol or PBS. Mice were injected with 0.5 mg/kg β-estradiol or PBS twice weekly. Tumor incidence **(B)** and tumor volume **(C)** were determined. **(D and E)** Serum IL-6 **(D)** and TNF-α **(E)** levels in mice were determined using a Bio-Plex cytokine panel (BioRad) and Luminex 200 (Luminex). **P < 0.01.

We investigated the effect of estrogen on W4P-induced tumor growth in male mice (Figure 6A). Treatment of male mice with β-estradiol drastically reduced the W4P-LHB-expressing tumor incidence. In the control group, six of eight mice developed tumor, whereas one of 10 mice developed tumor in the β-estradiol-treated group, and the tumor size was smaller than the average value in the PBS-treated group (Figure 6B, 6C). Four weeks after injection, the serum levels of IL-6 and TNF-α were determined. β-Estradiol significantly decreased serum IL-6 level by >60% compared with the PBS-treated group (Figure 6D). Serum TNF-α levels between the two nude mice groups did not differ significantly (Figure 6E). Taken together, our data strongly suggest that IL-6 plays a pivotal role in tumorigenicity and growth of W4P-expressing cells, and estrogen is capable of suppressing W4P-LHB-mediated tumorigenicity and tumor growth.

## Discussion

South Korea is still a high-endemic area for HBV infection, and HCC is more prevalent by 4.2–5.9 times in men than in women [28,29]. This male-to-female ratio of HCC incidence is higher than the average of 2.9:1 worldwide [30], which suggests that there are crucial viral factors in Korean chronic HBV patients favoring a male predominance. In an effort to determine HBV mutation types that contribute to gender disparity in HCC, we recently discovered the W4P/R mutation, which is found only in men, using a molecular epidemiological approach.

We selected W4P LHB and WT LHB from an HCC patient and a carrier with or without W4P mutation, respectively [27]. Sequence comparison between W4P LHB and WT LHB showed that there were seven variations including W4P. However, in our careful inspection of the literature, we did not find any notable mutations, except for W4P, related to liver disease severity. Thus, although the acquisition of W4P mutation is likely to affect mostly the tumorigenic nature of variant LHB, the synergistic or additive effect of other variations cannot be excluded.

In this study, we demonstrated that the expression of LHBs enhanced cell proliferation and W4P LHB exerted a stronger effect. Expression of W4P LHB resulted in downregulation of the p53 pathway. It is likely that W4P LHB impairs the cell-cycle checkpoint at the G1/S phase by inhibiting the p53–p21 axis, which in turn may contribute to HCC by the accumulation of mutations due to the maintenance of genome stability. Cyclin A plays an important role in the S and G2/M phases of the cell cycle [31]. It should be noted that W4P LHB upregulates expression of cyclin A in hepatocytes, because it is overexpressed in HCC tissue [32,33]. The enhanced colony-forming capability of W4P LHB provides further support for the potential role of the W4P mutation in HBV-related hepatocarcinogenesis. Similar patterns were observed in W4P-LHB-expressing Huh7 HCC cell lines. Taken together, our *in vitro* data suggest that mutation in the LHB region, such as W4P, during the natural course of chronic hepatitis B, may contribute to HCC generation. This strongly supports the previous epidemiological finding showing a higher prevalence of W4P mutation in patients with severe forms of liver disease than in those with milder forms of liver disease [27].

HBx contributes to gender disparity in HBV infection or HCC generation mainly due to signaling via the androgen axis [18,34,35]. However, although it is important for the initial replication in the host, it is not essential for HBV life cycle or chronic infection, so its function has been lost occasionally in chronic patients during the course of chronic hepatitis B via deletion events [34,36]. Therefore, another viral factor providing a likely explanation of gender disparity in HCC generation, mainly in the final stage of chronic hepatitis B, should be considered. Our *in vivo* mouse data clearly proved that W4P LHB, but not WT LHB, had higher potential for tumor formation in male than in female mice, suggesting mutations in LHB, such as W4P, occurring in a later stage of chronic hepatitis B, could contribute to the gender disparity in HCC generation.

Our *in vivo* data also showed that the gender disparity in W4P-LHB-induced tumorigenicity was closely related to the difference in IL-6 production between the genders. IL-6 is known to play a crucial role in the fibrosis and HCC related to liver regeneration [20,21,37]. Moreover, HCC patients with W4P variant displayed a higher IL-6 serum level than HCC patients with WT. The NIH3T3 cell line constitutively expressing W4P LHB induced preferential tumor formation in male nude mice over females, as well as having oncogenic potential at a high level, with 95% tumor generation incidence within 4 weeks. Our *in vivo* system showed a significant positive correlation between tumor size and IL-6 secretion, supporting previous reports regarding the positive role of IL-6 in tumorigenesis [20,21]. Estrogen was also shown to evoke biological defense against hepatocarcinogenesis by functioning as a negative regulator of IL-6 production in liver Kupffer cells [37]. In our study, we showed that estrogen suppressed production of IL-6 and subsequently reduced W4P-induced tumor growth in male mice, which suggests that a high level of estrogen in female mice suppresses tumorigenesis by W4P variant HBV through regulating IL-6 signaling. Thus, a likely explanation of the preponderance of tumor generation in male mice observed in our W4P-injected mouse model is that female hormonal factors play a suppressive role in W4P-induced tumorigenicity by inhibiting IL-6 production. Several studies have shown the relation between menopause and HCC risk in women. For example, a 1-year delay in the onset of menopause reduced the risk of HCC by 21%, and hormone replacement therapy was associated with a low risk of HCC [38,39]. Our data provide some insights and explanations for the gender disparity in HCC and how estrogen suppresses, at least in part, the tumorigenicity induced by HBV mutations.

## Conclusions

The novel HBV preS1 mutation W4P may contribute to HCC development in men with chronic hepatitis B in an IL-6-dependent manner. This is believed to be the first report showing direct involvement of HBV LHBs in gender disparity of tumorigenesis. This study provides a new insight into understanding the reason behind the male predominance of HCC in chronic hepatitis B patients. Our data showing an inhibitory effect of estrogen in tumor formation in male nude mice suggest that estrogen or estrogenic compounds have therapeutic potential in patients with W4P-LHB-induced HCC.

## Methods

### Generation of stable cell lines

Sequences encoding WT and variant LHBs were amplified from a patient with HBV carrier status and an HCC patient, which were proven without and with W4P/R mutation by a real-time polymerase chain reaction method as described previously [27]. Amplified products were cloned into the pIRES2 vector. NIH3T3 murine cell lines and Huh7 human HCC cell lines constitutively expressing the WT LHB and W4P variant were established by transfection with pIRES2-WT or pIRES2-W4P, followed by the selection with 500 μg/ml neomycin.

### Analysis of cell proliferation and cell cycle

To analyze the proliferation of cells, 10^4^ cells were seeded on 100-mm tissue culture dishes. The number of viable cells was determined at each time point by counting after trypan blue staining. To analyze the cell cycle, the DNA content of cells was assessed via propidium iodide staining, followed by flow cytometry. To analyze the role of JAK2 and stat3, cells were transfected with siRNAs targeting them (Bioneer, Daejeon, Korea) and subjected to conventional 3-(4, 5)-dimethylthiazol (−z-y1)-diphenyltetrazolium bromide (MTT) cell viability assay.

### Colony-forming assay

One hundred cells of each cell line were seeded in a six-well culture plate. After incubation for 14 days, colonies were fixed and stained with 0.5% methylene blue in ethanol for 10 min at room temperature. The number of cell colonies in each dish was counted under a microscope.

### Immunoblot analysis

Anti-preS1 (Aprogen, Daejeon, Korea), anti-cyclin A, anti-cyclin D1, anti-cdk2, anti-cdk4, anti-PCNA (Abcam, Cambridge, UK), anti-p53 and anti-β-actin (Santa Cruz Biotechnology Santa Cruz, CA, USA) antibodies were used for immunoblotting.

### ELISA

The amounts of secreted IL-6 and TNF-α were determined by mIL-6 and TNF-α ELISA kits (eBioscience, San Diego, CA, USA). The cells were incubated for 48 h and the supernatants were subjected to ELISA. To investigate the effect of estradiol on secretion of IL-6, cell lines were treated with 20 nM β-estradiol for 48 h. To examine the secretion of cytokines by macrophages, J774A.1 cells were incubated in the presence or absence of W4P-LHB-expressing tumor homogenate (10 mg) for 48 h with or without 20 mM estrogen.

### *In vivo* tumorigenicity study

About 10^7^ NIH3T3, WT-LHB-NIH3T3 and W4P-LHB-NIH3T3 cells were injected subcutaneously into the right hind legs of 8-week-old nude mice. Tumor formation was monitored over a 4-week period. Tumor volumes were calculated using the following equation: length × (width)^2^ × 0.52. The resected tumor masses were fixed and processed in an alcohol–xylene series followed by paraffin embedding. For histological examination, sections were stained with hematoxylin and eosin or subjected to immunohistochemistry with anti-preS1 monoclonal antibodies. To examine the effect of estrogen on tumor growth, male mice were injected with β-estradiol (0.5 mg/kg) or PBS. One week later, mice were injected with W4P-LHB-NIH3T3 cells subcutaneously together with β-estradiol (0.5 mg/kg) or PBS injection. Mice were injected with β-estradiol (0.5 mg/kg) or PBS twice weekly. Serum IL-6 and TNF-α levels were determined using a Bio-Plex cytokine panel (BioRad, CA). All of the animal experiments were conducted following NIH guidelines for housing and care of laboratory animals and in accordance with the protocol approved by Institutional Animal Care and Use Committee (IAUAC) of Seoul National University College of Medicine (protocol number SNU-111025).

### Patients

Sera from 22 male HCC and liver cirrhosis patients with WT and W4P LHBs were subjected to multiplex cytokine measurement. Patients information was previously described [27]. The Ethical Committee of Seoul National University Hospital (IRB No. C-1007-021-322) approved this research protocol and waived the need for written informed consent because routine diagnostic data were analyzed anonymously (Additional file 5: Table S1).

### Statistical analysis

All ELISA and cell proliferation assays in this study were repeated at least three times, and the results were expressed as percentages, means ± SD, or as medians (range). Differences between categorical variables were analyzed using Fisher’s exact test or χ^2^ test. For continuous variables, Student’s *t* test was used when the data showed a normal distribution, or the Mann–Whitney *U* test was used when the data were not normally distributed. A value of *P* < 0.05 (two-tailed) was considered to be statistically significant.

## Additional files

Additional file 1: Figure S1. WT and W4P variant LHB DNAs were cloned from a HBV carrier and a HCC patient, respectively. The amino acid sequences of the preS1 region from the reference strain (genotype A, B, C, and D) were aligned. (B) NIH3T3 cells stably expressing WT and W4P LHBs were established. Expression of LHBs were confirmed by immunoblotting with an anti-preS1 antibody. Additional file 2: Figure S2. NIH3T3 (left) and Huh7 (right) cell lines stably expressing WT and W4P LHBs were established. Expression of LHBs were confirmed by immunoblotting with an anti-preS1 antibody. Additional file 3: Figure S3. Induction of cell transformation by LHBs. Huh7 cell lines expressing WT and W4P LHBs were subjected to a colony formation assay. Data represent means ± SD from three independent experiments. *** *P* < 0.001 vs vector cell line, 1-way t-test. Additional file 4: Figure S4. Phosphorylation of stat3 and expression of proliferation related proteins of vector, WT-LHB or W4P LHB Huh7 cell lines were analyzed by immunoblotting. Additional file 5: Table S1. Clinical factors of patients.

## Abbreviations

HBV: Hepatitis B virus
HBx: HBV X protein
HCC: Hepatocellular carcinoma
IL-6: Interleukin-6
LHB: Large surface protein
TNF-α: Tumor necrosis factor-α
WT: Wild type
