# Supplementary material for: Male-specific hepatitis B virus large surface protein variant W4P potentiates tumorigenicity and induces gender disparity
Source: Mol Cancer. 2015 Feb 3;14(1):23. doi: 10.1186/s12943-015-0303-7 (PMC4326317; doi:10.1186/s12943-015-0303-7)
Supplement: Additional file 1: Figure S1. — WT and W4P variant LHB DNAs were cloned from a HBV carrier and a HCC patient, respectively. The amino acid sequences of the preS1 region from the reference strain (genotype A, B, C, and D) were aligned. (B) NIH3T3 cells stably expressing WT and W4P LHBs were established. Expression of LHBs were confirmed by immunoblotting with an anti-preS1 antibody. [file 12943_2015_303_MOESM1_ESM.pptx]

## Slide 1
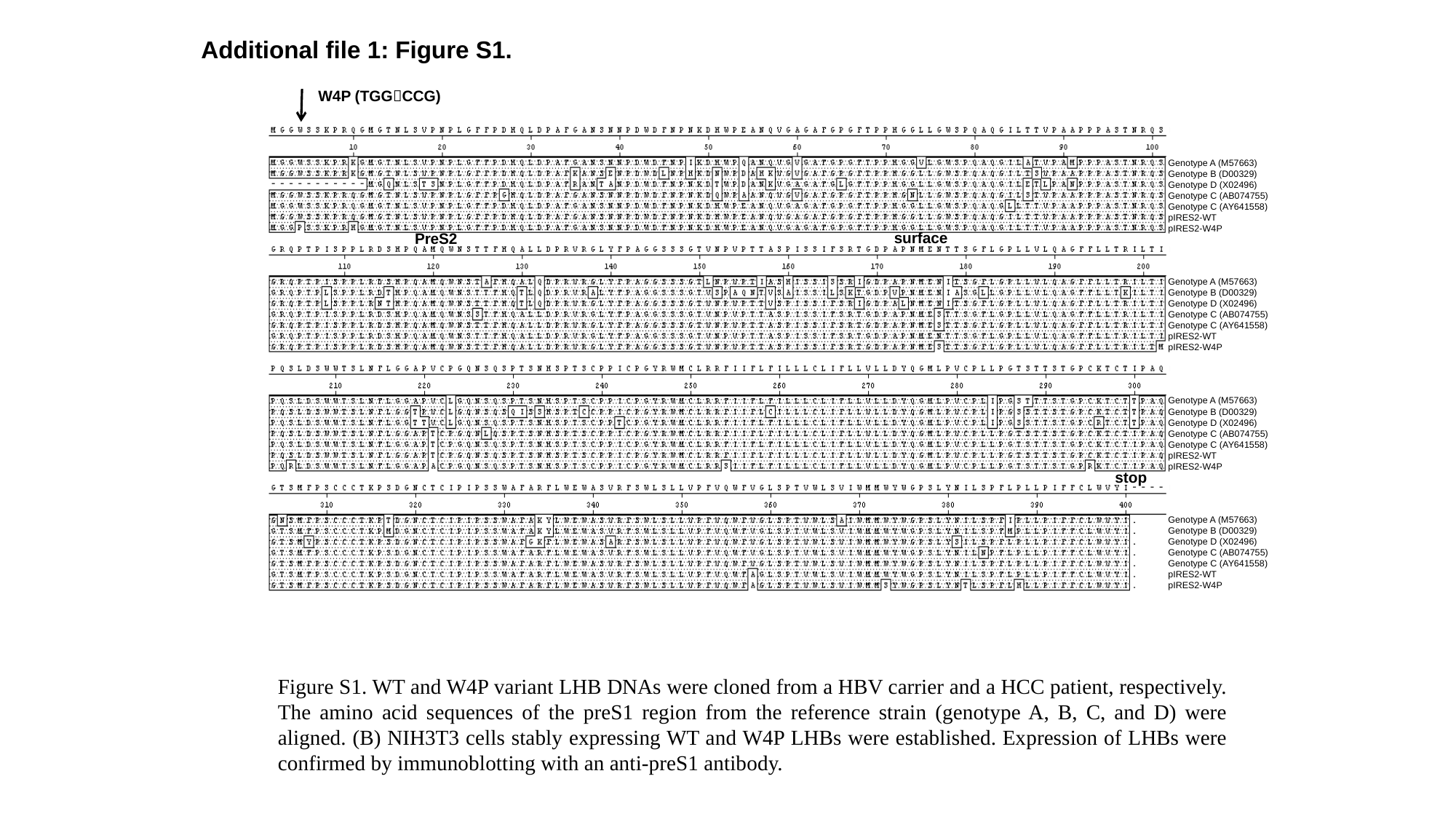

Additional file 1: Figure S1.
W4P (TGGCCG)
Genotype A (M57663)
Genotype B (D00329)
Genotype D (X02496)
Genotype C (AB074755)
Genotype C (AY641558)
pIRES2-WT
pIRES2-W4P
surface
PreS2
Genotype A (M57663)
Genotype B (D00329)
Genotype D (X02496)
Genotype C (AB074755)
Genotype C (AY641558)
pIRES2-WT
pIRES2-W4P
Genotype A (M57663)
Genotype B (D00329)
Genotype D (X02496)
Genotype C (AB074755)
Genotype C (AY641558)
pIRES2-WT
pIRES2-W4P
stop
Genotype A (M57663)
Genotype B (D00329)
Genotype D (X02496)
Genotype C (AB074755)
Genotype C (AY641558)
pIRES2-WT
pIRES2-W4P
Figure S1. WT and W4P variant LHB DNAs were cloned from a HBV carrier and a HCC patient, respectively. The amino acid sequences of the preS1 region from the reference strain (genotype A, B, C, and D) were aligned. (B) NIH3T3 cells stably expressing WT and W4P LHBs were established. Expression of LHBs were confirmed by immunoblotting with an anti-preS1 antibody.
