# Supplementary material for: Male-specific hepatitis B virus large surface protein variant W4P potentiates tumorigenicity and induces gender disparity
Source: Mol Cancer. 2015 Feb 3;14(1):23. doi: 10.1186/s12943-015-0303-7 (PMC4326317; doi:10.1186/s12943-015-0303-7)
Supplement: Additional file 2: Figure S2. — NIH3T3 (left) and Huh7 (right) cell lines stably expressing WT and W4P LHBs were established. Expression of LHBs were confirmed by immunoblotting with an anti-preS1 antibody. [file 12943_2015_303_MOESM2_ESM.pptx]

## Slide 1
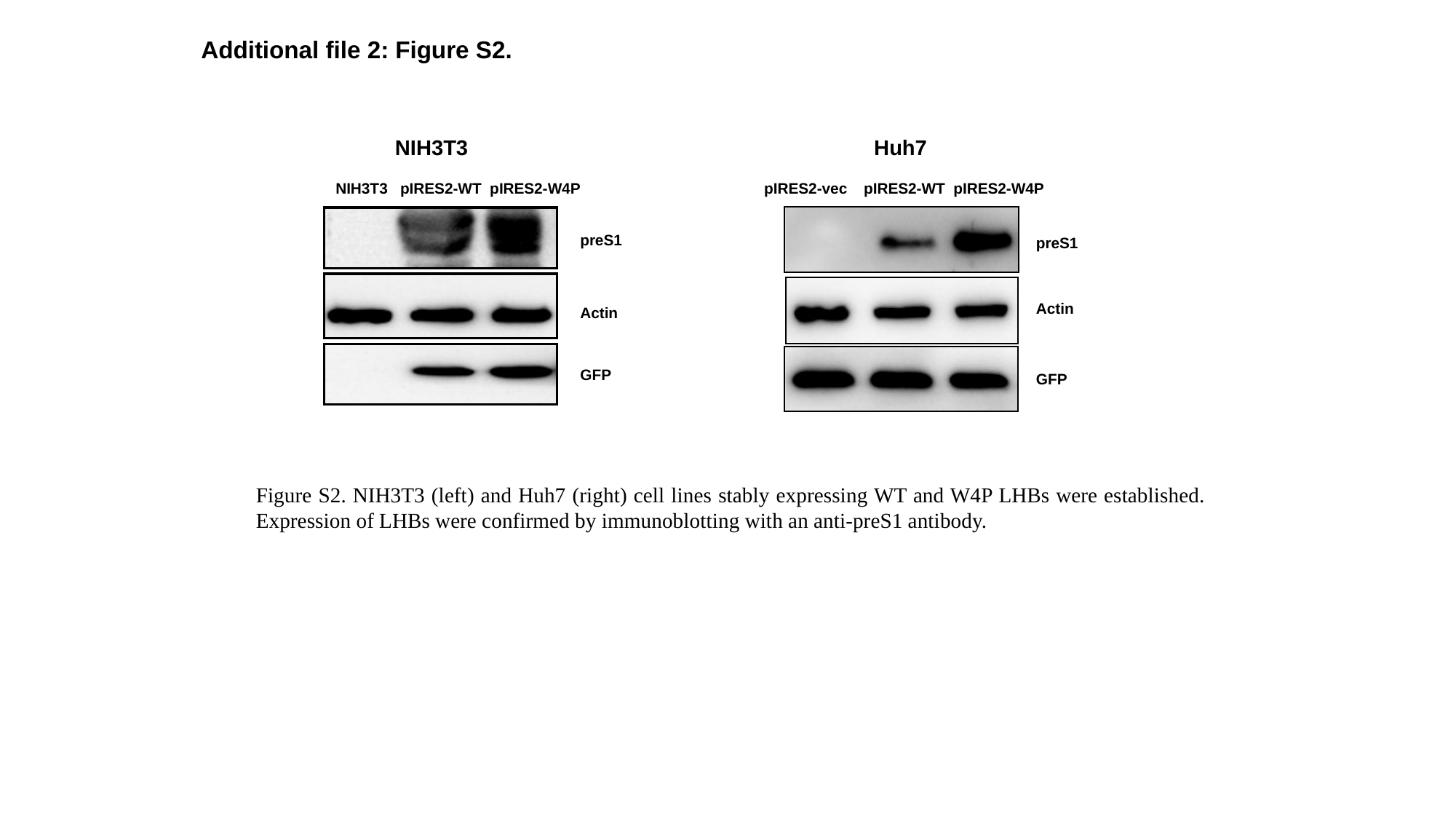

Additional file 2: Figure S2.
NIH3T3
Huh7
NIH3T3 pIRES2-WT pIRES2-W4P
pIRES2-vec pIRES2-WT pIRES2-W4P
preS1
preS1
Actin
Actin
GFP
GFP
Figure S2. NIH3T3 (left) and Huh7 (right) cell lines stably expressing WT and W4P LHBs were established. Expression of LHBs were confirmed by immunoblotting with an anti-preS1 antibody.
