# Supplementary material for: Male-specific hepatitis B virus large surface protein variant W4P potentiates tumorigenicity and induces gender disparity
Source: Mol Cancer. 2015 Feb 3;14(1):23. doi: 10.1186/s12943-015-0303-7 (PMC4326317; doi:10.1186/s12943-015-0303-7)
Supplement: Additional file 3: Figure S3. — Induction of cell transformation by LHBs. Huh7 cell lines expressing WT and W4P LHBs were subjected to a colony formation assay. Data represent means ± SD from three independent experiments. *** P < 0.001 vs vector cell line, 1-way t-test. [file 12943_2015_303_MOESM3_ESM.pptx]

## Slide 1
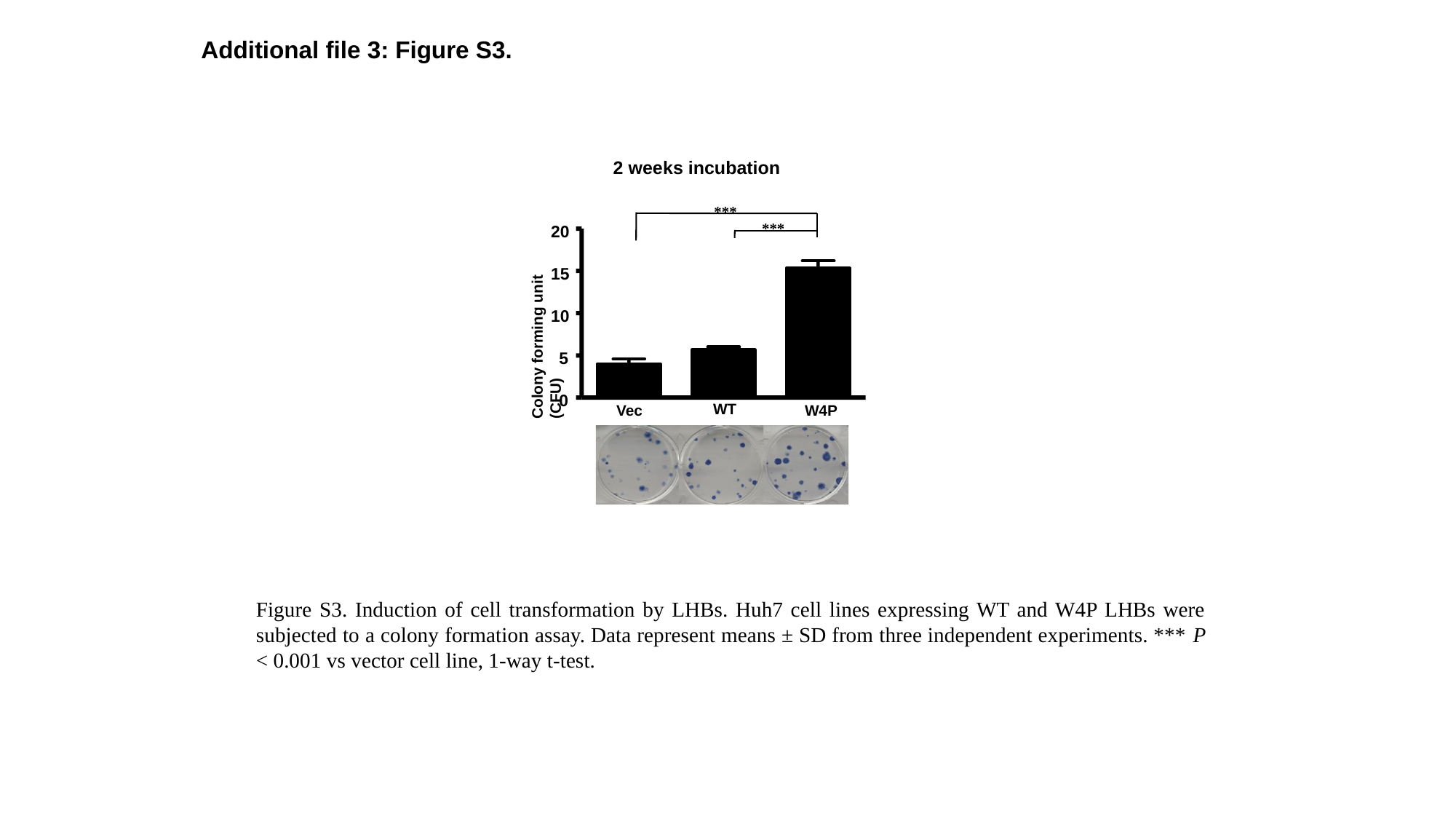

Additional file 3: Figure S3.
2 weeks incubation
20
15
10
5
0
***
***
Colony forming unit (CFU)
WT
Vec
W4P
Figure S3. Induction of cell transformation by LHBs. Huh7 cell lines expressing WT and W4P LHBs were subjected to a colony formation assay. Data represent means ± SD from three independent experiments. *** P < 0.001 vs vector cell line, 1-way t-test.
