# Supplementary material for: Male-specific hepatitis B virus large surface protein variant W4P potentiates tumorigenicity and induces gender disparity
Source: Mol Cancer. 2015 Feb 3;14(1):23. doi: 10.1186/s12943-015-0303-7 (PMC4326317; doi:10.1186/s12943-015-0303-7)
Supplement: Additional file 4: Figure S4. — Phosphorylation of stat3 and expression of proliferation related proteins of vector, WT-LHB or W4P LHB Huh7 cell lines were analyzed by immunoblotting. [file 12943_2015_303_MOESM4_ESM.pptx]

## Slide 1
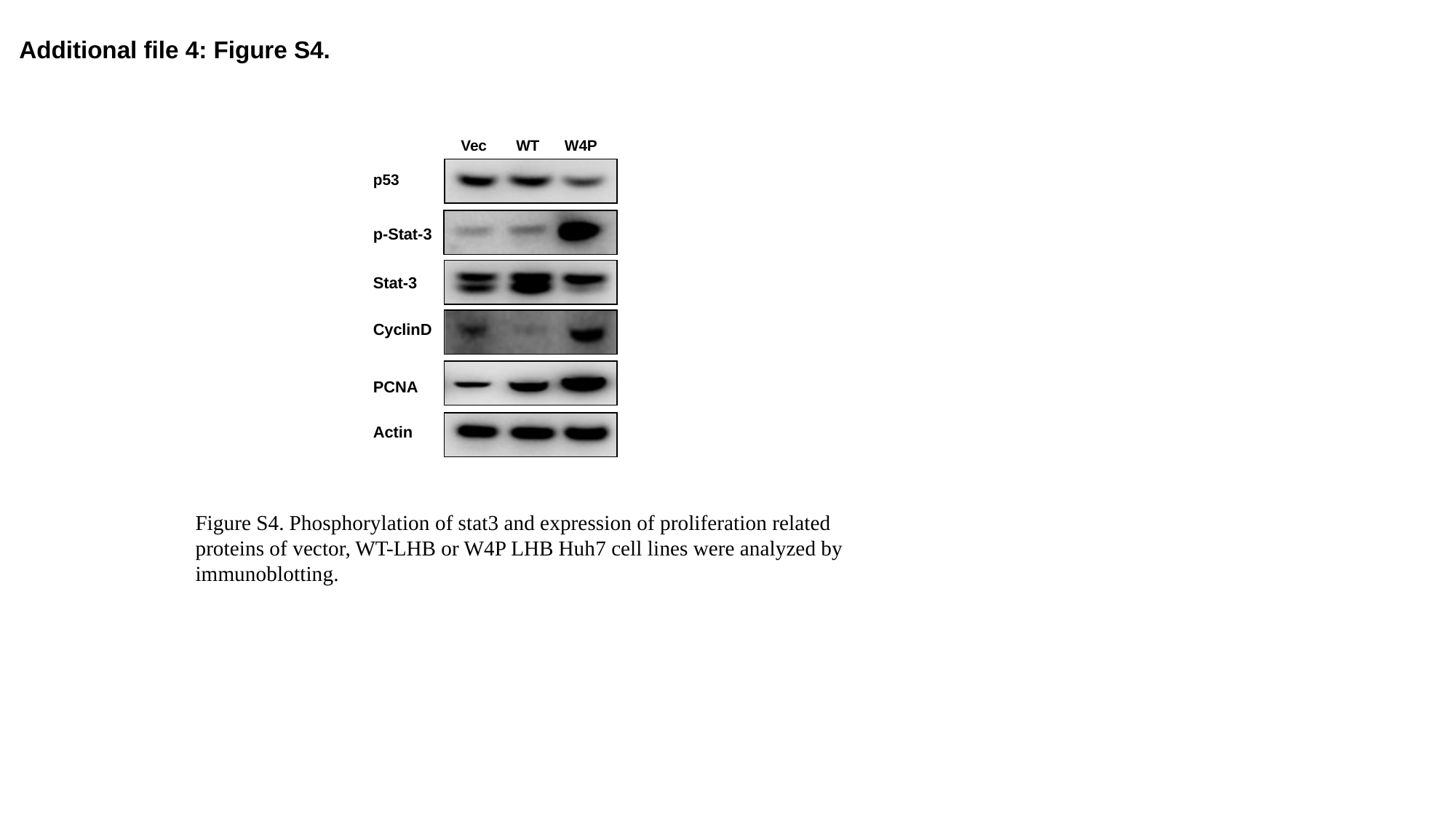

Additional file 4: Figure S4.
Vec WT W4P
p53
p-Stat-3
Stat-3
CyclinD
PCNA
Actin
Figure S4. Phosphorylation of stat3 and expression of proliferation related proteins of vector, WT-LHB or W4P LHB Huh7 cell lines were analyzed by immunoblotting.
