# Supplementary material for: Male-specific hepatitis B virus large surface protein variant W4P potentiates tumorigenicity and induces gender disparity
Source: Mol Cancer. 2015 Feb 3;14(1):23. doi: 10.1186/s12943-015-0303-7 (PMC4326317; doi:10.1186/s12943-015-0303-7)
Supplement: Additional file 5: Table S1. — Clinical factors of patients. [file 12943_2015_303_MOESM5_ESM.pptx]

## Slide 1
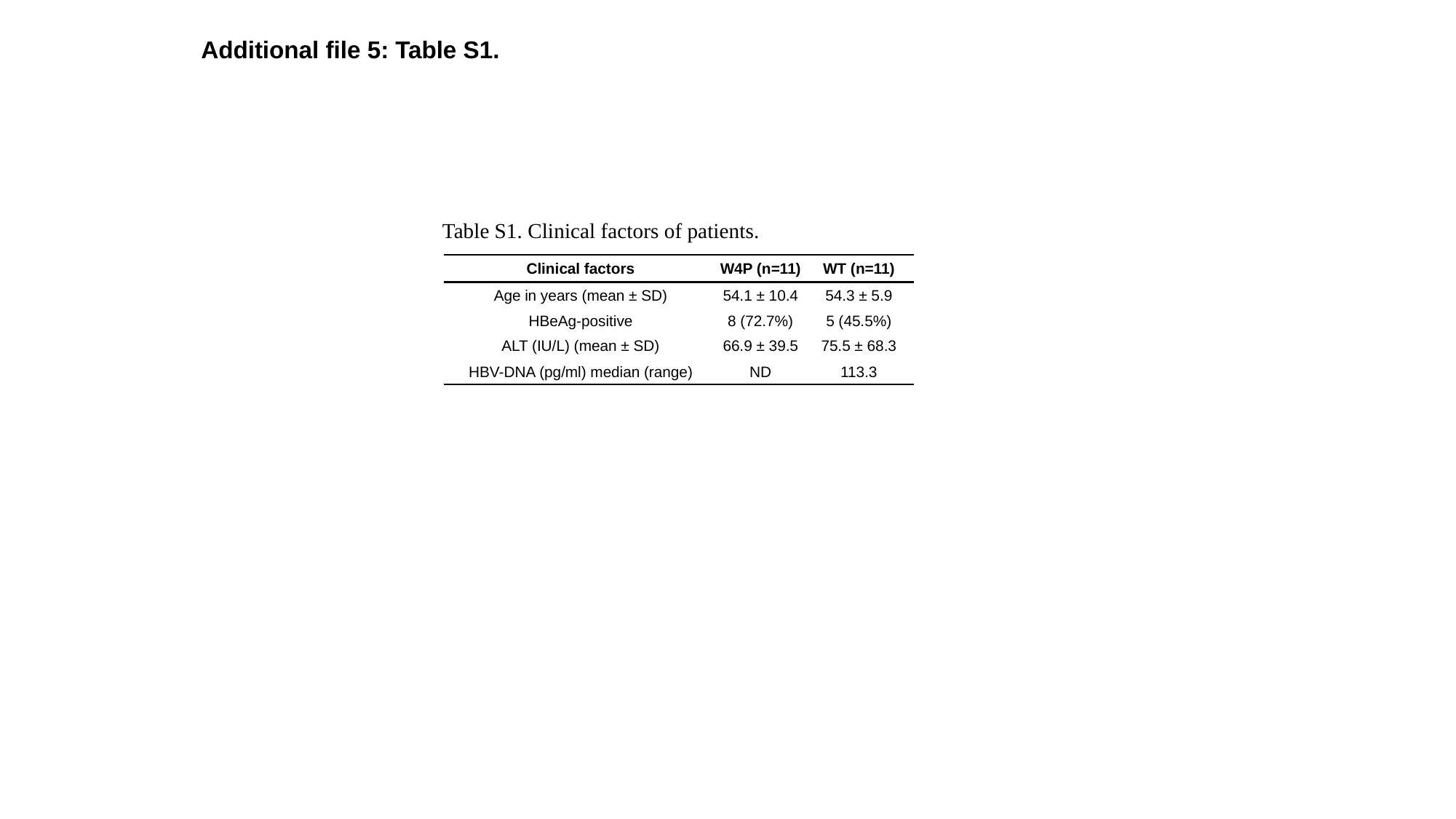

Additional file 5: Table S1.
Table S1. Clinical factors of patients.
| Clinical factors | W4P (n=11) | WT (n=11) |
| --- | --- | --- |
| Age in years (mean ± SD) | 54.1 ± 10.4 | 54.3 ± 5.9 |
| HBeAg-positive | 8 (72.7%) | 5 (45.5%) |
| ALT (IU/L) (mean ± SD) | 66.9 ± 39.5 | 75.5 ± 68.3 |
| HBV-DNA (pg/ml) median (range) | ND | 113.3 |
